# Metastatic Pheochromocytoma and Paraganglioma: Somatostatin Receptor 2 Expression, Genetics, and Therapeutic Responses

**DOI:** 10.1210/clinem/dgad166

**Published:** 2023-03-22

**Authors:** Alessa Fischer, Simon Kloos, Umberto Maccio, Juliane Friemel, Hanna Remde, Martin Fassnacht, Christina Pamporaki, Graeme Eisenhofer, Henri J L M Timmers, Mercedes Robledo, Stephanie M J Fliedner, Katharina Wang, Julian Maurer, Astrid Reul, Kathrin Zitzmann, Nicole Bechmann, Gintarė Žygienė, Susan Richter, Constanze Hantel, Diana Vetter, Kuno Lehmann, Hermine Mohr, Natalia S Pellegata, Martin Ullrich, Jens Pietzsch, Christian G Ziegler, Stefan R Bornstein, Matthias Kroiss, Martin Reincke, Karel Pacak, Ashley B Grossman, Felix Beuschlein, Svenja Nölting

**Affiliations:** Department of Endocrinology, Diabetology and Clinical Nutrition, University Hospital Zurich (USZ), and University of Zurich (UZH), CH-8091 Zurich, Switzerland; Department of Endocrinology, Diabetology and Clinical Nutrition, University Hospital Zurich (USZ), and University of Zurich (UZH), CH-8091 Zurich, Switzerland; Department of Pathology and Molecular Pathology, University Hospital Zurich, CH-8091 Zurich, Switzerland; Department of Pathology and Molecular Pathology, University Hospital Zurich, CH-8091 Zurich, Switzerland; Department of Internal Medicine I, Division of Endocrinology and Diabetes, University Hospital, University of Würzburg, 97080 Würzburg, Germany; Department of Internal Medicine I, Division of Endocrinology and Diabetes, University Hospital, University of Würzburg, 97080 Würzburg, Germany; Department of Internal Medicine III, University Hospital Carl Gustav Carus, Technische Universität Dresden, 01307 Dresden, Germany; Department of Internal Medicine III, University Hospital Carl Gustav Carus, Technische Universität Dresden, 01307 Dresden, Germany; Division of Endocrinology, Department of Internal Medicine, Radboud University Medical Center, 6525 GA Nijmegen, Netherlands; Hereditary Endocrine Cancer Group, Spanish National Cancer Research Center (CNIO), Madrid, Spain; Centro de Investigación Biomédica en Red de Enfermedades Raras, Madrid, Spain; First Department of Medicine, University Medical Center Schleswig-Holstein, 23538 Lübeck, Germany; Department of Medicine IV, University Hospital, Ludwig-Maximilians-University Munich, 80336 Munich, Germany; Department of Medicine IV, University Hospital, Ludwig-Maximilians-University Munich, 80336 Munich, Germany; Department of Endocrinology, Diabetology and Clinical Nutrition, University Hospital Zurich (USZ), and University of Zurich (UZH), CH-8091 Zurich, Switzerland; Department of Medicine IV, University Hospital, Ludwig-Maximilians-University Munich, 80336 Munich, Germany; Institute of Clinical Chemistry and Laboratory Medicine, University Hospital Carl Gustav Carus, Medical Faculty Carl Gustav Carus, Technische Universität Dresden, Fetscherstrasse, 01307 Dresden, Germany; Institute of Clinical Chemistry and Laboratory Medicine, University Hospital Carl Gustav Carus, Medical Faculty Carl Gustav Carus, Technische Universität Dresden, Fetscherstrasse, 01307 Dresden, Germany; Institute of Clinical Chemistry and Laboratory Medicine, University Hospital Carl Gustav Carus, Medical Faculty Carl Gustav Carus, Technische Universität Dresden, Fetscherstrasse, 01307 Dresden, Germany; Department of Endocrinology, Diabetology and Clinical Nutrition, University Hospital Zurich (USZ), and University of Zurich (UZH), CH-8091 Zurich, Switzerland; Department of Internal Medicine III, University Hospital Carl Gustav Carus, Technische Universität Dresden, 01307 Dresden, Germany; Department of Visceral and Transplantation Surgery, University Hospital, 8091 Zürich, Switzerland; Department of Visceral and Transplantation Surgery, University Hospital, 8091 Zürich, Switzerland; Institute for Diabetes and Cancer, Helmholtz Zentrum München, 85764 Neuherberg, Germany; Institute for Diabetes and Cancer, Helmholtz Zentrum München, 85764 Neuherberg, Germany; Department of Biology and Biotechnology, University of Pavia, 27100 Pavia, Italy; Department of Radiopharmaceutical and Chemical Biology, Institute of Radiopharmaceutical Cancer Research, Helmholtz-Zentrum Dresden-Rossendorf (HZDR), Dresden, Germany; Department of Radiopharmaceutical and Chemical Biology, Institute of Radiopharmaceutical Cancer Research, Helmholtz-Zentrum Dresden-Rossendorf (HZDR), Dresden, Germany; Faculty of Chemistry and Food Chemistry, School of Science, Technische Universität Dresden, Dresden, Germany; Department of Internal Medicine I, Division of Endocrinology and Diabetes, University Hospital, University of Würzburg, 97080 Würzburg, Germany; Department of Internal Medicine III, University Hospital Carl Gustav Carus, Technische Universität Dresden, 01307 Dresden, Germany; Department of Endocrinology, Diabetology and Clinical Nutrition, University Hospital Zurich (USZ), and University of Zurich (UZH), CH-8091 Zurich, Switzerland; Department of Internal Medicine III, University Hospital Carl Gustav Carus, Technische Universität Dresden, 01307 Dresden, Germany; Department of Medicine IV, University Hospital, Ludwig-Maximilians-University Munich, 80336 Munich, Germany; Department of Medicine IV, University Hospital, Ludwig-Maximilians-University Munich, 80336 Munich, Germany; Section on Medical Neuroendocrinology, Eunice Kennedy Shriver National Institute of Child Health and Human Development, National Institutes of Health, Rockville, MD 20847, USA; Green Templeton College, University of Oxford, Oxford, UK; NET Unit, ENETS Centre of Excellence, Royal Free Hospital, London, UK; Department of Endocrinology, Diabetology and Clinical Nutrition, University Hospital Zurich (USZ), and University of Zurich (UZH), CH-8091 Zurich, Switzerland; Department of Medicine IV, University Hospital, Ludwig-Maximilians-University Munich, 80336 Munich, Germany; Department of Endocrinology, Diabetology and Clinical Nutrition, University Hospital Zurich (USZ), and University of Zurich (UZH), CH-8091 Zurich, Switzerland; Department of Medicine IV, University Hospital, Ludwig-Maximilians-University Munich, 80336 Munich, Germany

**Keywords:** metastatic pheochromocytoma/paraganglioma, *SDHB* mutation, *SDHx* mutation, somatostatin receptor 2, somatostatin receptor–based therapies, PRRT

## Abstract

**Context:**

Pheochromocytomas and paragangliomas (PPGLs) with pathogenic mutations in the succinate dehydrogenase subunit B (*SDHB)* are associated with a high metastatic risk. Somatostatin receptor 2 (SSTR2)–dependent imaging is the most sensitive imaging modality for *SDHB*-related PPGLs, suggesting that SSTR2 expression is a significant cell surface therapeutic biomarker of such tumors.

**Objective:**

Exploration of the relationship between SSTR2 immunoreactivity and SDHB immunoreactivity, mutational status, and clinical behavior of PPGLs. Evaluation of SSTR-based therapies in metastatic PPGLs.

**Methods:**

Retrospective analysis of a multicenter cohort of PPGLs at 6 specialized Endocrine Tumor Centers in Germany, The Netherlands, and Switzerland. Patients with PPGLs participating in the ENSAT registry were included. Clinical data were extracted from medical records, and immunohistochemistry (IHC) for SDHB and SSTR2 was performed in patients with available tumor tissue. Immunoreactivity of SSTR2 was investigated using Volante scores. The main outcome measure was the association of SSTR2 IHC positivity with genetic and clinical–pathological features of PPGLs.

**Results:**

Of 202 patients with PPGLs, 50% were SSTR2 positive. SSTR2 positivity was significantly associated with *SDHB*- and *SDHx*-related PPGLs, with the strongest SSTR2 staining intensity in *SDHB*-related PPGLs (*P* = .01). Moreover, SSTR2 expression was significantly associated with metastatic disease independent of *SDHB/SDHx* mutation status (*P* < .001). In metastatic PPGLs, the disease control rate with first-line SSTR-based radionuclide therapy was 67% (n = 22, n = 11 *SDHx*), and with first-line “cold” somatostatin analogs 100% (n = 6, n = 3 *SDHx*).

**Conclusion:**

SSTR2 expression was independently associated with *SDHB/SDHx* mutations and metastatic disease. We confirm a high disease control rate of somatostatin receptor–based therapies in metastatic PPGLs.

Pheochromocytomas and paragangliomas (together, PPGLs) are rare endocrine tumors with a high degree of heritability (1). Pheochromocytomas originate from neural crest–derived cells of the adrenal medulla, while paragangliomas originate from paraganglia of the sympathetic (thorax, abdomen, pelvis) or parasympathetic (head and neck) nervous system.

Approximately 10% to 15% of pheochromocytomas and 35% to 40% of paragangliomas develop metastases (2-4). The latest World Health Organization classification defines PPGL-related malignancy by the presence of distant metastases at sites where chromaffin cells are physiologically absent, such as in bones or lymph nodes (5). Around 30% to 35% of all patients with PPGLs harbor germline mutations, while a further 35% to 40% harbor somatic mutations (6-8). According to their gene expression profile, PPGLs are divided into 3 main molecular clusters: (1) pseudohypoxia cluster (1A and lb), (2) kinase-signaling cluster, and (3) Wnt signaling cluster. Cluster 1 is further divided into cluster 1A (mutations in the Krebs cycle associated genes: *SDHA[AF2]*/*B*/*C*/*D*, *FH*, *MDH2*, *IDH*, *GOT2*, *SLC25A11*, and *DLST*) and 1B (mutations in the hypoxia-signaling pathway: *PHD1*/*2*, *VHL*, *HIF2A*/*EPAS1*, *IRP1*). Assignment to a specific molecular cluster is associated with differences in biochemical phenotype, clinical behavior, and long-term prognosis (3, 9).

PPGLs with mutations in succinate dehydrogenase subunit B (*SDHB)* belong to cluster 1A and are associated with a higher risk of metastatic disease than other hereditary PPGLs (3, 10, 11). Immunohistochemistry (IHC) allows the assessment of SDHB protein expression and has been shown to be absent in PPGLs with *SDHB* mutations (12). Therefore, screening for the absence of SDHB expression by immunostaining is a cost-effective method to identify patients likely to carry *SDHx* mutations (13).

The somatostatin receptors (SSTRs) 1 to 5 are G-protein–coupled receptors that bind somatostatin with high affinity (14). SSTRs are expressed throughout the central and peripheral nervous system as well as in various endocrine and neuroendocrine tissues and tumors (14, 15). Nonmetastatic as well as metastatic PPGLs have been shown to express somatostatin receptor subtype 2 (SSTR2) (15-17).

In clinical practice, SSTR2 functional imaging with somatostatin receptor analog (SSA)–positron emission tomography–computed tomography (PET/CT) is frequently used for the diagnosis and localization of PPGLs. Gallium-68-labeled [^68^Ga]-DOTA-SSA PET/CT has a sensitivity of 94% to 100% when used in the diagnosis and screening of PPGLs (11, 16, 18-20). Specifically, [^68^Ga]-DOTA-SSA PET/CT was shown to have a superior detection rate in *SDHx*-related PPGLs (*SDHx: SDHA*, *SDHB*, *SDHC*, *SDHD*) than ^18^F-FDG PET/CT and ^123^I-meta-iodobenzylguanidine (^123^I-MIBG) scintigraphy (18, 21-23). Somatostatin peptide receptor–based radionuclide therapy is currently a first-line systemic therapy options (although not officially approved in many countries) for SSTR2-positive metastatic PPGLs (3, 11, 18, 19, 24, 25). Peptide receptor–based radionuclide therapy is already an officially approved and highly effective therapy option for patients with advanced midgut and pancreatic neuroendocrine tumors (26).

In order to better define the subgroup of PPGL patients who may benefit from treatment with SSTR-based therapies, this retrospective study investigated the association of SSTR2 immunoreactivity with *SDHB* immunoreactivity, mutational status, and metastatic disease, and, additionally, evaluated the response of metastatic PPGLs (mPPGLs) with or without known germline and/or somatic mutations to SSTR-based therapies.

## Material and Methods

### Study Population

In this retrospective study, 285 patients with PPGLs treated at 6 specialized endocrine tumor centers in Germany (Munich: 95 patients; Dresden: 44 patients; Lübeck: 1 patient; Würzburg: 62 patients), The Netherlands (Nijmegen: 31 patients), and Switzerland (Zurich: 52 patients) were included. Patients were selected based on participation in the ENSAT registry (27) and the availability of medical records. Analysis of SSTR2 and SDHB immunoreactivity was performed in 202 PPGL tumors with available tumor tissue collected for previously described tissue microarrays (28). For analysis of treatment responses, 78 patients with metastatic PPGLs without available tumor tissue were additionally analyzed. Metastatic PPGL was defined by the presence of distant metastases at sites where chromaffin cells are physiologically absent (5). The study was conducted according to the law and regulations of the Cantonal Ethics Committee Zurich under the reference number BASEC 2017-00771 as part of ENS@T (European Network for the Study of Adrenal Tumors). Written informed consent was obtained from each patient, or in the case of children parental consent, prior to participation. Clinical data were extracted from medical records and treatment responses evaluated according to RECIST criteria when detailed reports were available.

The treatment history of patients who received SSTR-based therapies as first- or second-line therapies was further analyzed. The response to PRRT was evaluated 1 year after treatment initiation or considered as progressive disease when another therapy line was started within 1 year of treatment with PPRT. The response to treatment with “cold” somatostatin analogs was assessed 3 months after treatment initiation at the earliest from medical records. When no radiologic reports were available, responses to treatment and progression-free survival were used as described in “tumor board” decision letters. Therapies analyzed as part of this study were administered between August 2010 and March 2022.

### Immunohistochemistry

For 202 patients with available tumor tissue collected for previously described tissue microarrays (28), we performed IHC for SSTR2 and SDHB expression in the Department of Pathology at University Hospital Zurich.

Formalin-fixed, paraffin-embedded 4-μm-thick sections from tumor tissue were stained by IHC using anti-SSTR2A polyclonal antibody (clone SP44, dilution 1:25, Zytomed systems, Berlin, Germany; RRID:AB_2864701), anti-Ki-67 monoclonal antibody (clone SP6, dilution 1:100; Cell Marque Lifescreen Ltd; RRID:AB_1158037), and anti-SDHB monoclonal antibody (clone 21A11AE7, dilution 1:200, Abcam Limited; RRID:AB_2864701). Staining was performed with an automated immunostainer (DiscoveryUltra, Roche Ventana for SSTR2A staining and Ki-67 staining, Bond Leica Biosystems for SDHB staining) and visualized with OptiView DAB Kit.

#### Evaluation of SSTR2/SDHB immunohistochemistry

**Figure 1.**
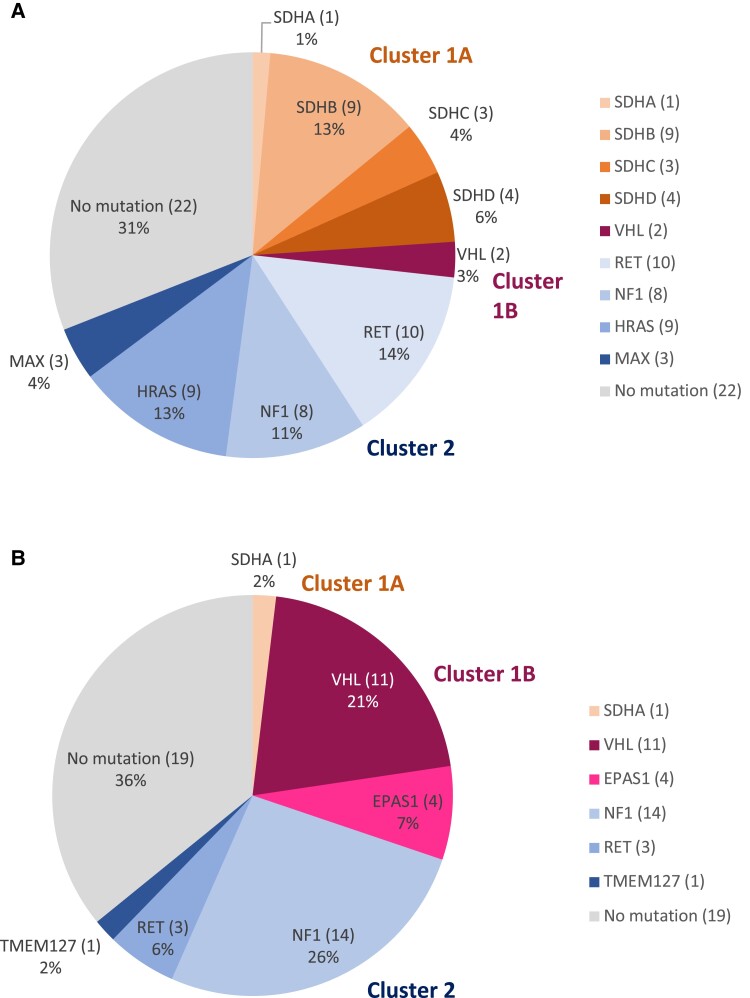
(A) Mutational landscape of n = 71 SSTR2 IHC-positive PPGLs according to clusters. Tumors without mutations detected in germline or somatic sequencing are depicted under “No mutation”. (B) Mutational landscape of n = 53 SSTR2 IHC-negative PPGLs according to clusters. Tumors without mutations detected in germline or somatic sequencing are depicted under “No mutation.”

Somatostatin receptor type 2A (SSTR2A) expression was semiquantitatively investigated using Volante scores (29). Volante scores range from 0 to 3 and take into account the subcellular localization and extent of the staining as follows: score 0: absence of immunoreactivity; score 1: pure cytoplasmic immunoreactivity, either focal or diffuse; score 2: membranous reactivity in less than 50% of tumor cells, irrespective of the presence of cytoplasmic staining; score 3: circumferential membranous reactivity in more than 50% of tumor cells, irrespective of the presence of cytoplasmic staining (29) (Figure 1 (30)). Samples with Volante scores 2 and 3 were considered positive for SSTR2, and scores 0 and 1 as negative.

SDHB IHC was scored ranging from 0 to 3 as described by Papathomas et al (13) as follows: score 0: negative as completely absent staining; score 1: weak diffuse as a cytoplasmic blush with no granularity; score 2: heterogeneous defined by granular cytoplasmic staining in combination with cytoplasmic blush but lacking defined diffuse granularity; score 3: positive granular cytoplasmic staining.

### Sequencing

Sequencing was performed by the local centers or by the Spanish National Cancer Research Center (CNIO) in Madrid, Spain in germline or tumor DNA by next-generation sequencing (NGS). Germline testing from patient blood samples was performed as part of routine clinical practice at the respective center, as described by Murakami et al (28). In order to detect somatic variants, NGS was performed with a custom multigene panel covering 84 genes (Gieldon et al (31)) or the human comprehensive cancer panel (Qiagen, DHS-3501Z) covering 306 cancer-associated genes, as previously described (32). At CNIO, somatic testing using frozen samples or formalin-fixed, paraffin-embedded samples was performed using NGS panels. Briefly, the targeted gene panel was designed using the TruSeq Custom Amplicon 1.5 kit system (Illumina, San Diego, CA). Those genes covered in the panel were the following: *VHL*, *RET*, *SDHA*, *SDHB*, *SDHC*, *SDHD*, *SDHAF2*, *SDHAF1*, *MAX*, *HIF1A* (exon 12), *HIF2A* (exon 12), *TMEM127*, *HRAS*, *KRAS*, *NF1*, *GOT2*, *FH*, *MDH2*, *SLC25A11*, *DNMT3A* (exon 8), *DLST* (exon 14), *MERTK* (exon 17), *IDH1*, *IDH2* (exon 4), *CSDE1*, *EGLN1*, *EGLN2*, *BRAF* (exon 15), *MET* (exons 14-21), *FGFR1* (exons 12 and 14), *KIF1B*, *CDKN1B*, *MEN1*, *PTEN*, *H3F3a*, *ATRX*, plus the *TERT* promoter region. Mutations were confirmed by Sanger sequencing, and also assessed in peripheral blood DNA samples when available. Multiplex ligation-dependent probe amplification (MRC Holland) was performed for *VHL* and *SDHx* using blood DNA in those with clinical signs but negative NGS results. Standards and guidelines of the American College of Medical Genetics and Genomics and the Association for Molecular Pathology (33) were applied for the classification of identified variants.

### Statistical Analysis

The proportion of SSTR2 positivity was expressed as a percentage. Clinicopathological characteristics were compared between groups of patients with SSTR2-positive vs those with SSTR2-negative PPGLs, using the Pearson chi-square-test, Fisher's exact test, or multivariable analysis of variance, where appropriate (nominal or ordinal). To analyze SSTR2 IHC intensity, comparisons between groups were performed by 1-way analysis of variance followed by the Bonferroni multiple comparisons test. Disease control rate (DCR) was calculated as the percentage of patients who achieved a complete response, partial response, or stable disease as the best response to therapy with SSTR-directed therapy. Progression-free survival (PFS) was defined as the length of time in months between first cycle of PRRT or start of somatostatin analogs and progression of disease, as determined by imaging. The Kaplan–Meier method was used to calculate median PFS. All reported *P* values are 2-sided hypothesis tests conducted at the *P* < .05 level, and adjustments were made for multiple comparisons (Benjamin and Hochberg false discovery rate). Statistical analyses were performed using IBM SPSS Statistics 28.0 (IBM Corporation, Armonk, NY, USA).

## Results

### Patient Characteristics

**Figure 2.**
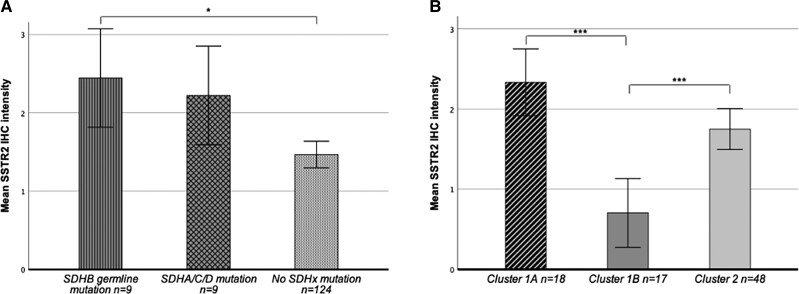
(A) Mean SSTR2 staining intensity in *SDHx-*related PPGLs compared with PPGLs without *SDHx* mutations. SSTR2 IHC staining intensity was scored semiquantitatively according to the Volante score (0 = absence of staining, 3 = strong staining). Statistical significance is denoted with stars (*P < .05). Error bars show standard error of the mean (SEM). (B) Mean SSTR2 staining intensity according to cluster. SSTR2 IHC staining intensity was scored semiquantitatively according to the Volante score (0 = absence of staining, 3 = strong staining). According to their mutation profile, PPGLs are divided into 3 main molecular clusters: (1) pseudohypoxia cluster (1A and 1B), (2) kinase-signaling cluster 2, and (3) Wnt signaling cluster 3. Cluster 1 is further divided into cluster 1A (mutations in the Krebs cycle associated genes) and 1B (mutations in the hypoxia-signaling pathway). Statistical significance is denoted with stars (***P < .001). Error bars show standard error of the mean (SEM).

A total of 285 patients with pheochromocytomas (211 patients) or paragangliomas (73 patients, plus 1 patient in which tumor entity not available) treated at 6 different European endocrine tumor centers were included in this study. Patient characteristics are summarized in Table 1. Germline and somatic mutations identified in our cohort are shown elsewhere (Table 1 (30)). The patient flow chart is shown elsewhere (Figure 2 (30)). *SDHx* and specifically *SDHB* mutations were associated with extra-adrenal disease, and germline *SDHB* mutations were associated with metastatic disease (*P* < .001). Tumor diameter greater than 5 cm was also associated with metastatic behavior (*P* < .001).

**Table 1.** Patient characteristics

	Nonmetastatic PPGL (n = 182)	Metastatic PPGL (n = 103)
Age at surgery		
Median (range)	51.0 (12-83)	48.6 (9-77)
Gender		
Female (%)	95 (52.2%)	40 (38.8%)
Male (%)	87 (47.8%)	63 (61.2%)
BMI*^a^*		
Median (range)	24.1 (16.7-44.3)	23.8 (19.9-43.3)
Diabetes*^a^*		
Yes	37 (20.3%)	12 (11.7%)
No	92 (50.5%)	45 (43.7%)
Unknown	53 (29.1%)	46 (44.7%)
Hypertension*^a^*		
Yes	122 (67.0%)	30 (29.1%)
No	29 (15.9%)	23 (22.3%)
Unknown	31 (17.0%)	50 (48.5%)
Sequencing performed		
Germline	25 (13.7%)	80 (77.6%)
Somatic	19 (10.4%)	2 (1.9%)
Germline and somatic	80 (44.0%)	5 (4.9%)
None	58 (31.9%)	16 (15.5%)
Mutation status		
Germline	33 (26.6%)	46 (52.9%)
Somatic	39 (31.5%)	4 (4.6%)
No mutation	34 (27.4%)	3 (3.4%)
No mutation by germline (no somatic sequencing)	18 (14.5%)	34 (39.1%)
Tumor location		
Pheochromocytoma	156 (85.7%)	55 (53.4%)
Paraganglioma	25 (13.7%)	48 (46.6%)
Not available	1 (0.5%)	0
Tumor size		
Maximal diameter, mean (±SD) in mm	44.5 (23.2)	64.7 (37.1)

Before primary resection of the tumor.

Abbreviations: BMI, body mass index; PPGL, pheochromocytoma and paraganglioma.

### Correlation of SSTR2 Expression With Clinical and Genetic Characteristics

IHC of SSTR2 was performed for 202 tumor samples including 25/202 (12.4%) metastatic cases: 101/202 (50.0%) showed positive staining for SSTR2 by IHC (strong SSTR2 staining: 35/202, 17.3%; moderate SSTR2 staining: 66/202, 32.7%) (Figure 1 (30)).

Variation in SSTR2 levels was not associated with tumor location. As such, there was no difference in SSTR2 levels between adrenal and extra-adrenal tumors nor between paragangliomas of the head or neck and abdomen (Table 2). Furthermore, SSTR2 levels were not associated with a higher proliferation index (Ki-67 ≥ 3%) nor with the size of the tumor.

**Table 2.** Characteristics of SSTR2 IHC-positive vs SSTR2 IHC-negative tumors

	SSTR IHC-positive PPGLs (n = 101)	SSTR IHC-negative PPGLs (n = 101)
Tumor location		
Pheochromocytoma	79 (78.2%)	87 (86.1%)
Paraganglioma (not head and neck)	10 (9.9%)	8 (7.9%)
Head and neck paraganglioma	11 (10.9%)	5 (5.0%)
Not available	1 (1%)	1 (1%)
Tumor size		
>5 cm	22 (21.8%)	35 (34.7)
<5 cm	56 (55.4%)	61 (60.4%)
Not available	23 (22.7%)	5 (5.0%)
Cluster*^a^*		
1A	17 (16.8%)	1 (1%)
1B	2 (2.0%)	15 (14.9%)
2	30 (29.7%)	18 (17.8%)
No mutation	22 (21.8%)	19 (18.8%)
Incomplete sequencing	30 (29.7%)	48 (47.5%)
Ki-67		
Ki-67 ≥ 3%	19 (18.8%)	20 (19.8%)
Ki-67 < 3%	73 (72.3%)	67 (66.3%)
Not available	9 (9.0%)	14 (13.9%)
SDHB IHC positive		
yes	82 (81.2%)	95 (94.1%)
No	14 (13.9%)	6 (5.9%)
Not available	5 (5.0%)	—

Abbreviations: IHC, immunohistochemistry; PPGL, pheochromocytoma and paraganglioma; SDHB, succinate dehydrogenase subunit B; SSTR, somatostatin receptor.

According to their mutation profile, PPGLs are divided into 3 main molecular clusters: (1) pseudohypoxia cluster (1A and 1B), (2) kinase-signaling cluster 2, and (3) Wnt signaling cluster 3. Cluster 1 is further divided into cluster 1A (mutations in the Krebs cycle associated genes) and 1B (mutations in the hypoxia-signaling pathway).

#### SSTR2 expression is associated with metastatic disease

In a multivariate analysis, SSTR2 IHC positivity was significantly associated with metastatic disease (*P* < .001), independently of germline *SDHB* mutations or tumor size. Metastatic PPGLs were SSTR2 IHC-positive in 23/25 (92.0%) cases compared with 78/177 (44.1%) nonmetastatic PPGLs (*P* < .001).

In the subgroup of 124 PPGLs without *SDHx* mutations, metastatic PPGLs were SSTR2 positive in 12/13 (92.3%) cases compared with 47/111 (42.3%) of nonmetastatic PPGLs (*P* < .001).

#### SDHB mutations are associated with SSTR2 IHC positivity and staining intensity

Germline or somatic sequencing data and SSTR2 IHC staining were available for 142 PPGLs. The mutational landscape of SSTR2 IHC positive and SSTR2 IHC negative PPGLs is shown in Fig. 1A and 1B. In total, 18/142 (12.7%) PPGLs harbored germline (n = 15) or somatic (n = 3) *SDHx* mutations. Of these, 9 had *SDHB* germline mutations, 4 had *SDHD* mutations (3 germline, 1 somatic), 3 had *SDHC* mutations (2 germline, 1 somatic), and 2 had *SDHA* mutations (1 germline, 1 somatic). Except for 1 germline *SDHA*-mutated PPGL, all *SDHx*-mutated PPGLs were SSTR2 IHC positive. *SDHx* mutations (*P* < .001) and *SDHB* germline mutations (*P* < .01) were significantly associated with SSTR2 IHC positivity. SSTR2 staining intensity was highest in *SDHB*-mutated PPGLs, and significantly higher in *SDHB*-related PPGLs (n = 9, mean 2.44, SD 0.52) than in PPGLs without *SDHx* mutations (including non-*SDHx*-related hereditary PPGLs) (n = 124, mean 1.47, SD 0.991) (*P* = .01) (Fig. 2A). SSTR2 IHC staining intensity was significantly higher in cluster 1A tumors (*SDHx* related, n = 18, mean 2.33, SD 0.59) compared with cluster 1B (n = 17, mean 0.71, SD 0.67) tumors (*P* ≤ .001), but not compared with cluster 2 tumors (n = 48, mean 1.75, SD 1.02) (Fig. 2B).

Even though the percentage of SSTR2-positive tumors in the subgroup of cluster 2–related PPGLs was lower than in cluster 1A–related PPGLs, all *HRAS*-related and *MAX*-related PPGLs were SSTR2 IHC positive. Metastatic disease was present in 3/9 (33.3%) of *HRAS*-related and 2/3 (66.6%) of *MAX*-related PPGLs.

SDHB protein levels by IHC were, however, not significantly associated with SSTR2 expression. SDHB staining was negative in 20/196 (10.2%) PPGLs. Of these 20 PPGLs, 14 harbored *SDHx* mutations, 3 had no mutations (germline sequencing only [n = 1], somatic sequencing [n = 2], with 1 displaying an *SDHA* variant of unknown significance), 1 harbored a somatic *VHL* mutation, while in 2 sequencing data were not available. Within this group of 20 SDHB-negative PPGLs, SSTR2 staining was negative in 6 PPGLs (1 SDHA related, 3 without mutation, 1 *VHL* mutated, and 1 without sequencing data).

### Retrospective Analysis of Therapy Response to SSTR-Based Therapy of Metastatic PPGLs

**Figure 3.**
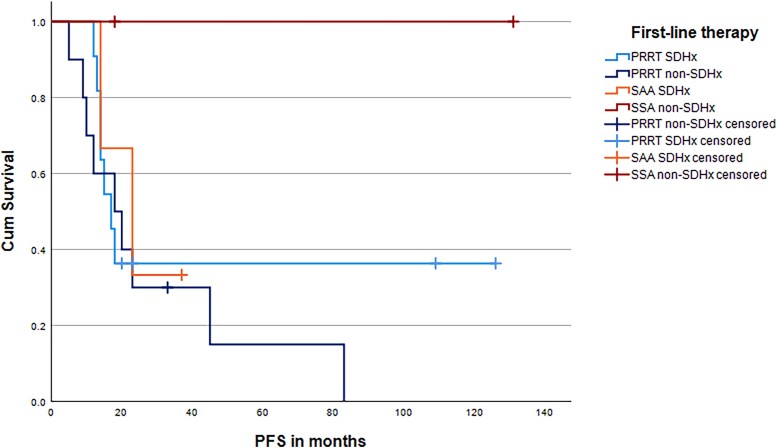
Kaplan–Meier curves for patients receiving SSTR-based therapies. Abbreviations: PRRT, somatostatin peptide receptor–based radionuclide therapy; SSA, somatostatin analogs.

Information on systemic treatment was available for 101/103 (98.1%) metastatic patients of the whole study cohort (n = 285). Of these, 33/101 (32.7%) were treated with first- or second-line SSTR2-based therapies (Table 3). PRRT was initiated as first-line therapy in 22/101 (21.8%) patients and as second-line therapy in 5/101 (5.0%) patients, respectively. Six patients (5.9%) were treated with “cold” somatostatin analogs (n = 3 lanreotide, n = 3 octreotide). The number of patients with progressive disease at baseline is shown in Table 4. DCR is presented in Table 3; median PFS under first-line and second-line therapy in months is presented in Table 5; Kaplan–Meier curves are shown in Fig. 3. Information on [^68^Ga]-DOTA-SSA PET/CT was available in 25/27 (92.6%) patients treated with PRRT and 3/6 (50%) patients treated with SSA (for the remaining 5 patients, information on imaging could not be retrieved from clinical records). [^68^Ga]-DOTA-SSA PET/CT was positive in all of these patients. In 6 patients treated with SSTR2-based therapies, SSTR2 IHC results were available (all SSTR2 IHC positive).

**Table 3.** Best response and DCR of systemic therapies with PRRT and SSA in mPGGLs

	First-line PRRT					Second-line PRRT					First-line SSA				
	PR	SD	PD	NA	DCR	PR	SD	PD	NA	DCR	PR	SD	PD	NA	DCR
**Mutation**															
*SDHA*	—	—	—	—	—	—	—	—	—	—	—	1	—	—	100%
*SDHB*	2	5	3 (1)	—	70%	—	1 (1)	1	—	50%	—	1	—		100%
*SDHD*	—	1 (1)	—	—	100%	—	—	—	—	—	—	1	—	—	100%
*HRAS*	—	—	1	—	0%	—	—	—	—	—	—	1 (1)	—		100%
*RET*	1	—	1	—	50%	—	—	—	—	—	—	—	—	—	—
No mutation	—	4	1 (1)	1 (1)	80%	—	1	1	—	50%	—	2	—	—	100%
NA	—	1	1	—	50%	—	1	—	—	100%	—	—	—	—	—
Total	3	11	7	1	67%	—	3	2	—	60%	—	6	—	—	100%

In brackets: number of SSTR2 IHC stained tumors (all stained tumors were SSTR2 IHC positive).

Abbreviations: DCR, disease control rate; NA, not available; PD, progressive disease; PR, partial response; PRRT, somatostatin peptide receptor–based radionuclide therapy; SSA, “cold” somatostatin analog; SD, stable disease.

**Table 4.** Number of patients with progression at baseline before first-line systemic therapy

	Progression at baseline			
Therapy	Yes	No	Not available	Total
PRRT	20 (9)	2 (2)	—	22 (11)
SSA	3 (2)	1 (1)	2	6 (3)

Number of patients with *SDHx-*mutations are shown in brackets.

Abbreviations: PRRT, somatostatin peptide receptor–based radionuclide therapy; SSA, Somatostatin analogs.

**Table 5.** PFS in months after first-line/second-line systemic therapy with PRRT or “cold” somatostatin analogs in mPPGL

	No. of patients		Median PFS in months		PFS lower limit In months		PFS upper limit in months	
	All*^a^*	*SDHx*	All*^a^*	*SDHx*	All*^a^*	*SDHx*	All*^a^*	*SDHx*
First-line PRRT	21	11	18	17	5	12	126	126
Second-line PRRT	5	2	18	11	11	11	95	20
First-line SSA	5	3	Not reached	Not reached	14	23	131	37

Abbreviations: PFS, progression-free survival; PRRT, somatostatin peptide receptor-based radionuclide therapy; SDH, succinate dehydrogenase; SSA, somatostatin analogs.

All patients including those with *SDHx* mutations.

#### PRRT

In 22 patients treated with first-line PRRT (20/22, 90% progressive at baseline), DCR was 67% (n = 10 *SDHB*, DCR 70%; n = 1 *SDHD*, stable disease; n = 11 *SDHB/D*, DCR 73%, 9/11 [81.2%] progressive at baseline). Overall, the PFS (n = 21) was 18 months (n = 10 *SDHB*, n = 1 *SDHD*, PFS 17 months).

Five patients received second-line PRRT (after MIBG [n = 2], after CVD chemotherapy [n = 2], after SSA [n = 1]) with a DCR of 60% (n = 2 *SDHB*, DCR 50%). The median PFS for PRRT as second-line treatment (n = 5) was 18 months (n = 2 *SDHB*, median PFS 11 months).

#### Somatostatin analogs

For 6 patients treated with first-line SSA (3/6 progressive at baseline), the DCR was 100% (*SDHA*, *SDHB*, *SDHD* related 1 patient each, DCR 100%); the median PFS with SSA was not reached (n = 5, n = 3 *SDHx*).

## Discussion

A key aspect of research in PPGLs is to identify biomarkers that distinguish benign from potentially metastatic PPGLs. Besides a larger diameter of the primary tumor (>5 cm) and the presence of a *SDHB* mutation (4, 5, 10, 34-36), we describe SSTR2 IHC positivity as a potential new independent marker of metastatic behavior. Leijon et al were unable to show an association of SSTR2 IHC positivity with metastatic behavior in 151 PPGLs (37). In contrast to our study with 25 metastatic PPGLs, the group of metastatic PPGLs in the above-mentioned study was relatively small (n = 14) and all metastatic paragangliomas (n = 10) were SSTR2 IHC positive but only 1/4 metastatic pheochromocytoma (37). Furthermore, mutation status was not known, and a scoring system other than Volante scores was used to classify immunoreactivity of SSTR2 (37). According to Volante et al, scores of 2 and 3 showed high concordance with SSTR scintigraphy (correlations of 87% and 94%, respectively, with SSTR scintigraphy, whereas scores 0 and 1 poorly correlated with SSTR results) (29).

We have also demonstrated that SSTR2 expression is significantly associated with *SDHB*-related PPGLs (100% SSTR2 positivity, highest staining intensity). Additionally, the subgroup of cluster 1A-related (*SDHx*-related) PPGLs shows a significantly higher percentage of SSTR2-positive tumors than the cluster 1B-related subgroup and than PPGLs without *SDHx* mutations. Previously, only a small number of studies reported the *SDHx* mutation status or SDHB protein loss by IHC together with the SSTR2 positivity by IHC (37, 38). In the study by Leijon et al (37), only 4/13 mPPGLs were *SDHB* mutated, and all were positive for SSTR2 by IHC.

In a study by Elston et al (38) of 182 PPGLs, 32 tumors were SDH deficient by immunohistochemistry, and SDH-deficient tumors were more likely to stain moderately or strongly for SSTR2A when compared with SDH-sufficient tumors (91% vs 49%, *P* < .0001, respectively) (38).

However, in our study, only the presence of an *SDHB/SDHx* mutation, but not loss of SDHB staining by IHC, was significantly associated with SSTR2 expression. Similar results were shown in the study by Leijon et al in a subgroup of 16 IHC SDHB-deficient PPGLs where IHC SDHB-negative tumors did not exhibit significantly higher SSTR2 positivity than SDHB-sufficient tumors (37). In our cohort, of the 20 IHC SDHB-deficient tumors 3 had no mutations (1 only by germline sequencing), 1 harbored a somatic *VHL* mutation, while for 2 IHC SDHB-deficient PPGLs, no sequencing was performed. These 6/20 IHC SDHB-deficient cases were IHC SSTR2 negative compared with 9/9 germline *SDHB*-mutated and 17/18 (94%) *SDHx*-mutated PPGLs with SSTR2 IHC positivity. This finding suggests that *SDHB/SDHx* mutations are specifically associated with SSTR2 expression. However, loss of SDHB protein expression itself may not be inevitably associated with SSTR2 protein expression, and indeed also occurs in PPGLs without *SDHB/SDHx* mutations. Of interest, all *MAX*-related (n = 2) and *HRAS*-related (n = 3) PPGLs in our study were also SSTR2 IHC positive. This finding suggests that SSTR2 expression is not exclusively associated with cluster 1A molecular pathways. However, the underlying molecular mechanism remains unclear.

Findings from preclinical studies using PPGL cell lines and tumor models may provide further leads toward the identification of SSTR2 regulatory mechanisms. One previous report on the effects of reactivated *EPAS1* expression in mouse pheochromocytoma cells in vitro provides evidence that HIF-2α downstream signaling may be involved in SSTR2 downregulation (39). These findings are in accordance with clinical reports demonstrating poor tumor detection rates for radiolabeled SSA in *EPAS1*-mutated PPGLs with impaired HIF-2α degradation (40). Furthermore, a recent animal study showed that SSTR2 levels can be stimulated in mouse pheochromocytoma allografts via treatment with epigenetic drugs (41).

###  

#### Retrospective analysis of therapy response

PRRT and cold SSTR2 analogs (octreotide/lanreotide), both targeting SSTRs on the cell surface, are therapeutic options for metastatic PPGLs (3, 11). Based on our finding that *SDHB/SDHx*-mutated PPGLs show highest SSTR2 positivity rates, we investigated therapy responses of mPPGLs to PRRT and somatostatin analogs according to the presence or absence of *SDHx* pathogenic variants.

#### PRRT

PRRT is a potential first-line therapeutic option for slowly to moderately growing mPPGLs with moderate to high tumor burden (3, 5, 42). The DCR with PRRT was reported as ≥80% in most previous studies (PFS 17-39 months) (19, 43-45, 45-55). In a retrospective study, the DCR with PRRT was 95% in *SDHB/SDHD*-mutated (n = 20) vs 93.8% in wild-type (n = 16) mPPGLs, with a significantly longer median PFS of the mutated group (not reached vs 51.5 months, *P* = .030) (48). In our study, the DCR with PRRT was slightly higher in the subgroup of *SDHB/D*-related PPGLs (73%) than the overall DCR (67%). Generally, the PFS and PFS of the *SDHB/D* subgroup was similar (18 months overall, *SDHB/SDHD* 17 months). The slightly lower DCR and PFS we found, compared with most previous studies, might be explained by mostly progressive disease at baseline (20/22, 90%) in our study. Considering that most patients were progressive at baseline, we found a comparatively high DCR and PFS with PRRT.

#### Somatostatin analogs (SSA)

Data from retrospective or prospective studies investigating SSA in PPGLs are lacking (25, 42). Only a small number of case reports on use of SSA in patients with paragangliomas have been published so far (56-58). However, based on the mechanism of action, PPGLs with high SSTR2 expression (such as *SDHB/SDHx*-related PPGLs) might be expected to respond well to SSA. This has already been shown for metastatic gastroenteropancreatic neuroendocrine tumors (59, 60). In patients with SSTR2-positive metastatic gastroenteropancreatic neuroendocrine tumors, SSA significantly prolonged the PFS (median PFS with lanreotide not reached vs placebo 18 months, estimated PFS lanreotide at 24 months 65.1% vs placebo 33.0%; median PFS octreotide long-acting release (LAR) 14.3 months vs placebo 6 months) (60, 61). A phase 2 study on the therapy of mPPGLs with the SSA lanreotide is currently recruiting (LAMPARA, NCT03946527).

In neuroendocrine tumor cells, the SSTR2 agonist octreotide reduced intracellular levels of VEGF by decreasing HIF-1α cell content (62). In PPGLs, *SDHx* mutations lead to a disruption of the Krebs cycle which leads via inactivation of PHD1/2, to lower HIF-α hydroxylation and thus cause less HIF-α degradation, and subsequently HIF-α accumulation (42). It could be hypothesized that SSTR2 agonists, as shown in neuroendocrine tumor cells, also decrease HIF-α content in PPGL cells and are therefore especially effective in PPGLs with disruption of the Krebs cycle such as *SDHx-*related PPGLs.

In our study, 6 patients (n = 3 *SDHx*) were treated with first-line SSA (n = 3 lanreotide, n = 3 octreotide), and showed an exceptionally high DCR and PFS (DCR 100%, median PFS not reached), including 3 patients with progression at baseline. Since SSAs are well tolerated, they may well represent a first-line therapy option for slowly progressing PPGLs if good efficacy is confirmed in larger studies such as LAMPARA. On an individual case-by-case decision, they may already be considered with good disease stabilization.

Our study has certain limitations, especially its retrospective nature. Radiology imaging reports from staging were not available for all patients. Therefore, assumptions about best response and date of progression had to be made based on patient history described in medical reports or tumor board recommendation letters. Furthermore, possible cross-reactivity of the SSTR2A antibody used in this study with other SSTR types cannot be fully excluded.

In conclusion, our study demonstrates that *SDHB* and *SDHx* mutations are significantly associated with SSTR2 expression, with the highest IHC staining intensity in *SDHB*-related PPGLs. Moreover, SSTR2 expression is associated with metastatic disease independent of *SDHB*/*SDHx* mutation status. Thus, SSTR2 IHC positivity might be a novel biomarker of potential metastatic behavior of PPGLs. Previous studies on metastatic phenotypes in pheochromocytoma allograft mice support this hypothesis (63). Furthermore, our study shows high DCRs with SSTR-based therapies in mPPGLs including the subgroup of *SDHx*-related PPGLs.

## Data Availability

Some or all datasets generated during and/or analyzed during the current study are not publicly available but are available from the corresponding author on reasonable request.

## Abbreviations

DCR: disease control rate
IHC: immunohistochemistry
NGS: next-generation sequencing
PET/CT: positron emission tomography–computed tomography
PFS: progression-free survival
PPGL: pheochromocytoma and paraganglioma
SDHB: succinate dehydrogenase subunit B
SSA: somatostatin receptor analog
SSTR: somatostatin receptor
